# Antimicrobial Effects and Active Compounds of the Root of *Aucklandia Lappa* Decne (Radix Aucklandiae)

**DOI:** 10.3389/fchem.2022.872480

**Published:** 2022-04-06

**Authors:** Xuewei Cai, Chunping Yang, Guangwei Qin, Min Zhang, Yan Bi, Xiaoyan Qiu, Liya Lu, Huabao Chen

**Affiliations:** College of Agronomy, Sichuan Agricultural University, Chengdu, China

**Keywords:** Radix Aucklandiae, antimicrobial activity, alantolactone, dehydrocostus lactone, costunolide

## Abstract

The development of new biological fungicides using plant metabolites has become an important direction for pesticide development, and previous studies found that Radix Aucklandiae had a certain inhibitory effect on plant pathogens. In this study, we systematically studied the antimicrobial activity of extracts of Radix Aucklandiae, and the active compounds were isolated, purified and structurally identified. Ethanol extracts of Radix Aucklandiae had different inhibitory effects on seven common plant-pathogenic fungi, with EC_50_ (concentration for 50% of maximal effect) values ranging from 114.18 mg/L to 414.08 mg/L. The extract at concentration of 1,000 mg/L had a significant control effect on strawberry grey mould and wheat powdery mildew of more than 90%. Three active compounds were isolated and purified from the extract, which were identified as alantolactone, dehydrocostus lactone and costunolide. All three compounds showed significant inhibitory effects on *Botrytis cinerea*, and the MIC (minimal inhibitory concentration) values were 15.63 mg/L, 3.91 mg/L and 15.63 mg/L. Dehydrocostus lactone also showed obvious inhibitory effect on *Fusarium graminearum* with an MIC value of 62.25 mg/L. The extract of Radix Aucklandiae has high antimicrobial activity against some common plant-pathogenic fungi, and the work lays a foundation for the development of extracts of Radix Aucklandiae as botanical fungicides.

## Introduction

Plant-pathogenic fungi can cause a decline in crop yield and quality directly or indirectly and secrete a variety of toxins and harmful metabolites (Guo et al., 2021). Therefore, it is necessary to control fungal diseases in agricultural production. At present, chemical fungicides are widely used to control plant diseases. However, chemical fungicide abuse has had a serious negative impact on human health and environmental safety, especially leading to prominent “3R” (Resistance, resurgence, and Residue) problems (Ju, 2011). Therefore, it is urgent to develop environmentally friendly fungicides. Botanical fungicides are pesticides used to control plant diseases with the advantages of high efficiency, low or no toxicity, easy degradation, high selectivity and a low risk of inducing drug resistance (Bhandari et al., 2021). Therefore, the development of new botanical fungicides is a hot spot in the development of new environmentally friendly pesticides. At present, many plant extracts have been proven to have antimicrobial activity. For example, extracts of *Sophora flavescens* have inhibitory effect on *Gibberella zeae*, *Glomerella cingulata* and *Botrytis cinerea* (Li et al., 2006). Extracts of *Syzygium aromaticum* (L.) have inhibitory effect on *Colletotrichum gloeosporioides* and *Fusarium oxysporum* f. sp. *cubense* (He et al., 2006).

Radix Aucklandiae (Chinese trade name: Muxiang) is the dried root of *Aucklandia lappa* Dence. (a perennial herb of the genus *Saussurea*, family Compositae), which is cultivated in Yunnan and Sichuan Provinces (Shu et al., 2015). It is widely used in clinical medicine because of its anti-inflammatory, anti-ulcer, hepatoprotective, cholagogic, antitumour and other functions (Bocca et al., 2004; Lai et al., 2008; Wang et al., 2008; He et al., 2011; Butturini et al., 2014; Sun et al., 2015). In addition, it has also been proven that Radix Aucklandiae has antimicrobial activity in agricultural production. The water extract of Radix Aucklandiae has inhibitory effect on *Botrytis cinerea* and *Alternaria alternata* (Hasi et al., 2009). Its ethanol extract also can inhibit *Penicillium italicum* Wehmer, *Verticillium dahlia*, *Fusarium oxysporium*, *Rhizoctonia solani* Kuhn and *Gloeosporium piperatum* (Hu et al., 2009; Liu et al., 2011; Wang et al., 2012; Jin et al., 2019). However, the antimicrobial activity compounds of Radix Aucklandiae is not clear and has not been systematically explored at present.

The inhibitory effect of extracts of Radix Aucklandiae on common plant-pathogenic fungi was systematically studied based on the above, and active compounds of extracts of Radix Aucklandiae were isolated, purified and identified, which laid a foundation for the further development and utilization of the extract as a fungicide.

## Materials and Methods

### Materials

Radix Aucklandiae was purchased from Bozhou Huakai Electronic Commerce Co., Ltd. The 11 common pathogens including *F. graminearum*, *Blumeria graminis* (*Bgt*), *B. cinerea*, *C. gloeosporioides*, *Sclerotinia sclerotiorum*, *F. oxysporum*, *Fusarium lateritium*, *A. alternata*, *Pythium aphanidermatum*, *D. glomerata* and *Phytophthora infestans* were preserved and provided by the College of Agronomy of Sichuan Agricultural University. The wheat (Triticum aestivum L.) variety Chuannong 30, which is mildew and is grown at the College of Agronomy of Sichuan Agricultural University.

### Preparation of Extracts of Radix Aucklandiae

Plant extracts were prepared by solvent extraction (Zhang and Wang, 2011; Chen et al., 2012). Radix Aucklandiae was dried in an electrothermal constant temperature blast drying oven at 55°C, crushed into dry powder with a tissue grinder and stored in a sealed fresh-storage bag away from light. Dry powder (2 kg) was extracted by soaking in 5 times volume ethanol while avoiding light. The extract was filtered out, and the same amount of ethanol was added after 3 days. The extract was filtered out after 2 days, and the two parts of the filtrate were combined. The filtrate was concentrated to paste at 50–60°C by a rotary evaporator and stored in a refrigerator at 4°C for later use.

### Toxicity Determination of Extracts of Radix Aucklandiae

The toxicity was determined by the mycelium growth rate (Wang et al., 2014). The paste extract was dissolved in dimethyl sulfoxide (DMSO) and then prepared into 1,000 mg/L, 500 mg/L, 250 mg/L, 125 mg/L, 62.5 mg/L, and 31.25 mg/L solutions with double distilled water (contain 0.1% Tween 80). The solution (1 ml) was mixed with Potato Dextrose Agar (PDA) medium (containing 1% streptomycin sulfate) (9 ml) and then poured into a sterile Petri dish (9 cm in diameter) to make a medium-filled plate. After the medium was solidified, an agar block (with a diameter of 0.5 cm) containing pathogenic fungi to be tested was placed in each medium plane, and the side of the agar block containing fungi was placed onto the surface of the medium. Each concentration was tested using three biological replicates. Double distilled water (containing 0.1% Tween 80 and 2% DMSO) was used as the negative control. Pathogenic fungi were cultured at 25°C. The diameter of colony growth was measured by the cross method after 2–7 days, and the inhibition rate of mycelium growth was calculated. Then, the toxicity regression equation and EC_50_ value of the extract were obtained according to the probit analysis method for toxicity.

Colony growth diameter(mm)=Average of measured diameters–5(diameter of agar block)

$$\text{M}\text{ycelium}\;\text{growth}\;\text{inhibition}\;\text{rate}\left(\%\right)=\frac{\text{C}\text{K}-\text{P}\text{T}}{\text{C}\text{K}}\times \mathrm{100}$$

where CK is the negative control colony growth diameter, and PT is the colony growth diameter under extract solution treatment.

### Bioassay of Extracts of Radix Aucklandiae *In Vivo*

#### Control Effect on Wheat Powdery Mildew

The paste extract was dissolved in DMSO and then prepared into 1,000 mg/L, 500 mg/L and 250 mg/L solutions with double distilled water (contain 0.1% Tween 80). Wheat seeds were planted in a glass tube (4 cm in diameter), and the tube was sealed with parafilm. Seeds were cultured to the three-leaf stage at 20 ± 1°C and 60–70% humidity with a 16:8 h light/dark photoperiod. Then, wheat leaves were sprayed with 250 mg/L, 500 mg/L and 1,000 mg/L extract solutions with three replications at each concentration. Leaves were also sprayed with 100 mg/L prothioconazole as a positive control and with double distilled water (contain 2% DMSO and 0.1% Tween 80) as a negative control. Fresh spores of *Bgt* were inoculated onto the plants by shaking over the foliage of the wheat seedlings (Xie et al., 2021). The protective activity was determined by spray application of the extract solution first followed by inoculation with the pathogenic fungi 24 h later; the curative activity was determined by inoculation with the pathogenic fungi first followed by spray application of the extract solution 24 h later. Protective activity and curative activity were reflected by relative disease control efficiency (RDCE). The methods for detecting the protective and curative activities of the subsequent experiment were the same. Wheat was further cultured after treatment, and the disease incidence was investigated 7, 9 and 11 days after treatment. The disease was divided into six grades according to the percentage of lesion area to leaf area (Grade 0: 0%; Grade 1: less than 5%; Grade 3: 6–10%; Grade 5: 11–20%; Grade 7: 21–50%; Grade 9: more than 51%) (Liu et al., 2000). Then disease index and RDCE was calculated using the following formula:

$$\text{Disease}\;\text{index}=\frac{\text{Σ}\left(\text{Number}\;\text{of}\;\text{diseased}\;\text{leaves}/\text{plants}\;\text{per}\;\text{grade}\times \text{Corresponding}\;\text{grade}\right)}{\text{Total}\;\text{number}\;\text{of}\;\text{diseased}\;\text{leaves}\times \text{9}}$$

$$\text{RDCE}\left(\text{\%}\right)=\left(\frac{\text{C}\text{K}-\text{P}\text{T}}{\text{P}\text{T}}\right)\times \text{100}$$

Where, CK is DI of sterile water-treatment; PT is DI of medicament treatment.

#### Control Effect on Wheat Head Blight

Wheat seeds were planted in pots (15 cm in diameter) and cultured to the earing and flowering stage at 26 ± 2°C and 65–75% humidity in the greenhouse. Then, wheat leaves were sprayed with 400 mg/L and 4,000 mg/L extract solutions with three replications at each concentration. Leaves were also sprayed with 100 mg/L tebuconazole as a positive control and with double distilled water (contain 2% DMSO and 0.1% Tween 80) as a negative control. A spore suspension of *F. graminearum* (10 μL) was injected into wheat panicles with a microinjector. Spores were observed and counted on a haemocytometer under an optical microscope (4 × 10). The method for adjusting the spore suspension in the later experiment was the same. It is advisable to adjust the spore concentration to 80–100 spores per field. Wheat was further cultured after treatment, and the disease incidence was investigated 7, 9 and 12 days after treatment. The disease was divided into five grades according to the percentage of dry ear area to ear area (Grade 0: 0%; Grade 1: less than 25%; Grade 3: 26–50%; Grade 5: 51–75%; Grade 7: more than 76%) (Zhu et al., 2007). Then the disease index and the RDCE was calculated using the formula in 4.2.1.

#### Control Effect on Strawberry Grey Mould

Fresh strawberry fruits of uniform size were selected, soaked in 75% ethanol for 2 min, washed with double distilled water and then dried. Then, fruits were sprayed with 500 mg/L and 1,000 mg/L extract solutions with three replications at each concentration. Leaves were also sprayed with 100 mg/L pyraclostrobin as a positive control and with double distilled water (contain 2% DMSO and 0.1% Tween 80) as a negative control. The equator of the strawberry was pricked with a sterile needle of a 1 ml injector, a 2 mm wound was formed, and then a suspension of *B. cinerea* was evenly spread on the fruit surface (Han et al., 2019). The fruit was placed in a culture plate and stored at 25°C and 95% humidity after treatment. The disease incidence was investigated 3, 5 and 7 days after treatment. The disease was divided into six grades according to the percentage of lesion area to fruit surface area (Grade 0: 0.0%; Grade 1: less than 5.0%; Grade 2: 5.1–15.0%; Grade 3: 15.1–30.0%; Grade 4: 30.1–50.0%; Grade 5: more than 50.1%) (Han et al., 2019), and then the disease index and the RDCE were calculated using the formula in 4.2.1.

#### Control Effect on Citrus Anthracnose

Fruits of Jincheng orange with consistent appearance and no mechanical damage were selected, soaked in 75% ethanol for 2 min, washed with double distilled water and then dried. Then, the fruits were sprayed with 2000 mg/L and 4,000 mg/L extract solutions. Three biological replicates were performed at each concentration with 10 fruits per treatment. Leaves were also sprayed with 86 mg/L pyraclostrobin as a positive control and with double distilled water (contain 2% DMSO and 0.1% Tween 80) as a negative control. *C. gloeosporioides* was inoculated by needle puncture. The depth of the pinhole was 2 mm, and a spore suspension (10 μL) was dropped at the pinhole (Tian et al., 2019). The fruits were cultured at 28°C and 95% relative humidity after treatment. The diameters of the lesions were measured by the cross method, and the mycelium growth inhibition rate 7 days after treatment was calculated using the formula in 2.3.

#### Data Processing and Analysis

The inhibition results of different concentrations of extracts of Radix Aucklandiae against different pathogenic fungi were recorded, and the data were statistically analysed with SPSS Statistics 23.

### Isolation, Purification and Structure Identification of Active Compounds From Radix Aucklandiae

#### Macroporous Resin Isolation

D101 macroporous resin was soaked in absolute ethanol for 24 h. Then, it was loaded into the chromatography column after the resin was fully swollen. The resin was rinsed repeatedly with absolute ethanol until the supernatant was free of white turbidity and then rinsed with distilled water until no ethanol was available. The paste extract of Radix Aucklandiae was evenly dispersed in double distilled water. Then, the treated solution (10 L) was adsorbed statically with D101 macroporous resin (2 kg) for 48 h. Then, resin was loaded into the chromatographic column. The resin was eluted with distilled water (3 times the volume of the column), and the eluent was discarded. Then, the resin was eluted with 70% ethanol (5 times the volume of the column). The eluent was collected and concentrated under pressure to obtain the crude extract of Radix Aucklandiae.

#### Chromatography Isolation

The crude extract was evenly dispersed in double distilled water. The aqueous solution was extracted with petroleum ether at the same volume 4 times. The organic phase was concentrated under reduced pressure at 45°C to obtain petroleum ether extract. The extract was isolated by normal-phase silica gel chromatography and then eluted with different petroleum ether and ethyl acetate mixtures (50:0, 10:1 and 1:1 by volume). Three fractions (Fr. H1-H3) were isolated and antimicrobial active fractions were traced and selected by the spore germination method using *B. cinerea* as an indicator pathogen. After further silica gel column chromatography isolation, the fraction Fr.H1-1 was obtained by eluting Fr.H1 with petroleum ether: acetone (15:1 by volume) as the eluent. In addition, the fractions Fr. H2-1 and Fr. H3-1 were obtained by eluting Fr.H2 and Fr. H3 with petroleum ether and ethyl acetate mixtures (15:1, 20:1 and 1:1 by volume) as the eluent.

#### Preparation of High-Performance Liquid Chromatography (HPLC)

Fractions (Fr. H1-1, Fr. H2-1 and Fr. H3-1) were further isolated through a Phenomenex C18 column (250 × 10 mm, Phenomenex, Aschaffenburg, Germany) and UV detector. Fr. H1-1 was eluted with 30% acetonitrile (containing 0.1% formic acid) to obtain compounds numbered Compound **1** (20 mg), Compound **2** (20 mg) and Compound **3** (5 mg). Fr. H2-1 was eluted with 35% acetonitrile (containing 0.1% formic acid) to obtain compounds numbered Compound **4** (20 mg) and Compound **5** (20 mg). Fr. H3-1 was eluted with 35% acetonitrile (containing 0.1% formic acid) to obtain compounds numbered Compound **6** (20 mg) and Compound **7** (20 mg). The antimicrobial active compound was traced and selected by the spore germination method using *B. cinerea* as indicator pathogen.

#### Compound Structure Identification

The purity was determined by HPLC. The mass spectrum, ^1^H-NMR (nuclear magnetic resonance) and ^13^C-NMR spectra of the compounds with higher purity were determined (the ^1^H-NMR spectra were 400 MHz; the ^13^C-NMR spectra were 100 MHz; and the solvent was CDCl_3_). The chemical structure of the compound was identified according to spectroscopic data.

#### Compound Activity Identification

The MIC against the pathogen spores was determined by the microtiter method (Yang et al., 2012). The pure compound was prepared with DMSO in mother liquor at 2000 mg/L. Then, mother liquor was diluted with double distilled water to nine concentrations: 1,000 mg/L, 500 mg/L, 250 mg/L, 125 mg/L, 62.25 mg/L, 31.125 mg/L, 15.625 mg/L, 7.8125 mg/L, 3.906 mg/L, 1.953 mg/L. Compound solutions of different concentrations (5 μL) and PDA (45 μL) were added to each well of a 96-well plate and fully mixed. Spore suspension (10 μL) was added to each well after standing for 10 min, and DMSO solution (10 μL) was added as a negative control. Three biological replicates were performed per treatment. The degree of conidial germination in all wells was observed to determine the MIC value after cultivation at 28°C for 48 h.

## Results

### Toxicity Determination of Extracts of Radix Aucklandiae

The extract of Radix Aucklandiae showed different degrees of inhibition on the mycelial growth of 10 different plant-pathogenic fungi (Table 1). Among them, the inhibitory effects of the extract on *B. cinerea*, *S. sclerotiorum*, *C. gloeosporioides*, *F. oxysporum*, *A. alternata*, *F. graminearum* and *D. glomerata* were significant, and the EC_50_ values were 114.18 mg/L, 142.40 mg/L, 251.87 mg/L, 299.34 mg/L, 315.07 mg/L, 398.74 mg/L and 414.08 mg/L, respectively. The extract had a poor inhibitory effect on *P. infestans*, *F. lateritium* and *P. aphanidermatum*, and the EC_50_ values were in the range of 500–1,000 mg/L.

**TABLE 1:** Toxicity test results of extracts of Radix Aucklandiae against several plant-pathogenic fungi.

Plant-pathogenic fungi	Toxicity regression equation	Correlation coefficient	EC_50_(mg/L)	Confidence intervals (mg/L)
*F. graminearum*	y = 1.0107x+5.4036	0.9839	398.74	210–750
*B. cinerea*	y = 3.6591x+8.4484	0.9800	114.18	90–140
*C. gloeosporioides*	y = 0.7907x+5.4735	0.9786	251.87	80–800
*S. sclerotiorum*	y = 3.8796x+8.2840	0.9927	142.40	120–170
*F. oxysporum*	y = 1.7997x+5.9428	0.8997	299.34	230–400
*F. lateritium*	y = 0.7094x+5.1638	0.9081	587.69	310–1,120
*A. alternata*	y = 2.0376x+6.0220	0.9503	315.07	250–400
*P. aphanidermatum*	y = 1.3650x+5.645	0.9018	757.71	530–1,090
*D. glomerata*	y = 1.6341x+5.6257	0.8786	414.08	260–670
*P. infestans*	y = 1.0780x+5.2852	0.9804	543.82	350–830

### Bioassay of Extracts of Radix Aucklandiae *In Vivo*

#### Control Effect on Wheat Powdery Mildew

The different concentrations of extracts of Radix Aucklandiae had different effects on the control of wheat powdery mildew (Table 2). With the increase in the concentration of the extract, the control effect on wheat powdery mildew was higher, and the protective activity was better than the curative activity. When the extract concentrations were 1,000 mg/L, the RDCEs of protective and curative acivities were higher than that of the control agent (100 mg/L propiconazole).

**TABLE 2:** RDCE of extracts of Radix Aucklandiae on wheat powdery mildew.

Treatment	Concentration (mg/L)	Protective activity			Curative activity		
		7d (%)	9d (%)	11d (%)	7d (%)	9d (%)	11d (%)
Extracts of Radix Aucklandiae	1,000	96.86ab	91.69b	90.53b	90.53b	78.28b	57.11c
	500	86.15c	84.29c	83.42c	83.42c	70.85c	55.81c
	250	61.19d	57.89d	56.07d	56.07d	35.04d	32.56d
Prothioconazole	100	92.31b	84.53c	80.32c	88.62bc	76.35b	70.46b

Statistical significance was determined using one-way ANOVA. Different lowercase letters in the footnote in the same column showed significant difference in the relative disease control efficiency of different treatment at each time points (*p* < 0.05).

#### Control Effect on Wheat Head Blight

Different concentrations of extracts of Radix Aucklandiae had different degrees of control effects on wheat head blight (Table 3). The higher the concentration, the better the control effect, and the protective activity was better than the curative activity, but the control effect of the extract was lower than that of the control agent (86 mg/L Tebuconazole). When the concentration of extracts was 4,000 mg/L, the protective and curative activities of wheat head blight were the best. The RDCEs of the protective activities were 56.26%, 51.98 and 11.90%, and of curative activities were 40.63, 36.39, 11.90% at 7, 9 and 12 days after treatment, respectively.

**TABLE 3:** RDCE of extracts of Radix Aucklandiae on wheat head blight.

Treatment	Concentration (mg/L)	Protective activity			Curative activity			
		7d (%)	9d (%)	12d (%)	7d (%)		9d (%)	12d (%)
Extracts of Radix Aucklandiae	400	37.51c	11.96c	4.76c	34.38c		27.30c	2.38c
	4,000	56.26b	51.98b	11.90b	40.63b		36.39b	11.90b
Tebuconazole	86	71.88a	81.99a	88.10a	54.56a		87.50a	80.95a

Statistical significance was determined using one-way ANOVA. Different lowercase letters in the footnote in the same column showed significant difference in the relative disease control efficiency of different treatment at each time points (*p* < 0.05).

#### Control Effect on Strawberry Grey Mould

Different concentrations of extracts of Radix Aucklandiae had different degrees of control effects on strawberry grey mould (Table 4). When the concentration of the extract was 500 mg/L, it had obvious protective and curative activitys on strawberry gray mold. The RDCEs of the protective activities were 100.00, 85.71 and 77.78%, and those of the curative activities were 100.00, 75.00, 63.64% at 3, 5 and 7 days after treatment, respectively. When the concentration of extract was 1,000 mg/L, the RDCE of protective and curative activities reached 100%, which can achieve the control effect of the control agent (100 mg/L pyraclostrobin).

**TABLE 4:** RDCE of extracts of Radix Aucklandiae on strawberry grey mould *in vivo*.

Treatment	Concentration (mg/L)	Protective activity			Curative activity		
		3d (%)	5d (%)	7d (%)	3d (%)	5d (%)	7d (%)
Extracts of Radix Aucklandiae	1,000	100.00a	100.00a	100.00a	100.00a	100.00a	100.00a
	500	100.00a	85.71b	77.78b	100.00a	75.00b	63.64b
Pyraclostrobin	100	100.00a	100.00a	100.00a	100.00a	100.00a	100.00a

Statistical significance was determined using one-way ANOVA. Different lowercase letters in the footnote in the same column showed significant difference in the relative disease control efficiency of different treatment at each time points (*p* < 0.05).

#### Control Effect on Citrus Anthracnose

Different concentrations of extracts of Radix Aucklandiae had different degrees of control effects on citrus anthracnose (Table 5). The higher the concentration, the better the control effect, and the protective activity was better than the therapeutic effect, but the control effect of the extract was lower than that of the control agent (86 mg/L Tebuconazole). When the concentration of the extract was 4,000 mg/L, the protective and curative activities on citrus anthracnose were the best. The inhibitory rate for the protective activity was 67.65%, and that of the protective activity was 57.5% at 7 days after treatment.

**TABLE 5:** Control effect of extracts of Radix Aucklandiae on citrus anthracnose *in vivo* at 7 days after treatment.

Treatment	Concentration (mg/L)	Protective activity		Curative activity	
		Average diameters of lesions (cm)	Inhibitory rate (%)	Average diameters of lesions (cm)	Inhibitory rate (%)
Extracts of Radix Aucklandiae	4,000	0.55	67.65b	0.68	57.50b
	2000	0.80	52.94c	0.93	38.54c
	1,000	1.12	34.31d	1.17	27.08d
Tebuconazole	86	0.52	69.61a	0.54	66.15a
CK	-	1.70	-	1.60	-

CK is negative control. Statistical significance was determined using one-way ANOVA. Different lowercase letters in the footnote in the same column showed significant difference in the inhibitory rate (*p* < 0.05).

### Chemical Structure Identification of Active Compounds From Radix Aucklandiae

#### Compound 2

**FIGURE 1:**
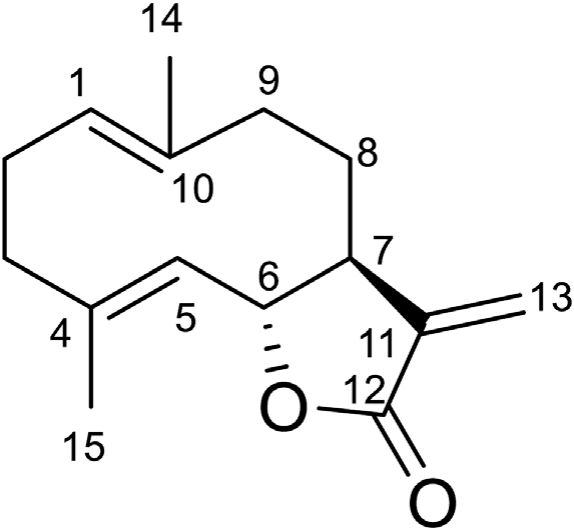
Chemical structural of costunolide.

Compound **2** was obtained as white amorphous powder. Its molecular formula was determined to be C_15_H_20_O_2_ based on the HRESIMS data (m/z 233.1540 [M + H]^+^), indicating six degrees of unsaturation. The ^1^H-NMR data (Table 6) showed four olefinic protons at *δ*_H_ 6.24 (1H, d, *J* = 3.6 Hz), 5.51 (1H, d, *J* = 3.2 Hz), 4.83 (1H, m) and 4.72 (1H, d, *J* = 9.9 Hz); one oxygen-bearing methine signal at *δ*_H_ 4.55 (1H, dd, 8.8, 9.8), a methine signal at *δ*_H_ 2.55 (1H, m), and two methyl proton signal at *δ*H 1.68 (3H, d, *J* = 1.3 Hz), 1.40 (3H, s). The ^13^C-NMR spectrum (Table 6) contained fifteen carbon signals, which were assigned to one lactone carbonyl carbon (*δ*c 170.6), six olefinic carbons (*δ*_C_ 141.4, 140.0, 136.9, 127.2, 127.0, 119.6), one oxy-methine carbon (*δ*_C_ 81.9), one methine carbon (*δ*_C_ 50.3), four methylenes (*δ*_C_ 40.9, 39.4, 28.0, 26.1), and two methyl carbons (*δ*c 17.3, 16.0). These spectral data were elucidated to the published data of Costunolide (Kaur et al., 2017; Chacon-Morales et al., 2020). Therefore, compound **2** was identified as costunolide and its molecular formula is shown in Figure 1. MS diagrams and ^1^H-NMR and ^13^C-NMR spectra were shown in Figures S1, S2 and S3, respectively.

**TABLE 6:** ^1^H and ^13^C-NMR data of Compound 2, Compound 5 and Compound 6 (CDCl_3_, *δ* in ppm, *J* in Hz).

No.	Compound 2^a^		Compound 5^a^		Compound 6^a^	
	*δ*_H_	*δ*_C_	*δ*_H_	*δ*_C_	*δ*_H_	*δ*_C_
1	4.83 (m)	127.1	2.89 (m)	47.7	2.03 (m)	41.9
2	2.08 (m)	28.1	1.89 (m)	32.7	2.07 (m)	16.9
3	1.81 (m)	41.1	2.51 (m)	30.4	2.43 (m)	32.8
4	-	140.2	-	151.4	2.23 (m)	37.7
5	4.72 (d, 9.9)	127.4	2.89 (m)	52.1	-	149.2
6	4.55 (dd, 8.8, 9.8)	82.0	3.96 (t, 9.2)	85.4	5.14 (d, 4.1)	118.9
7	2.55 (m)	50.5	2.89 (m)	45.2	3.56 (m)	39.6
8	1.70 (m)	26.3	2.15 (m); 1.41 (m)	31.1	4.81 (dt, 3.0, 6.5)	76.6
9	1.46 (m)	39,5	2.51 (m); 2.23 (m)	36.4	2.55 (m)	42.8
10	-	137.1	-	149.4	-	140.0
11	-	141.6	-	139.9	-	32.8
12	-	170.6	-	170.4	-	170.6
13	6.24 (d, 3.4); 5.51 (d, 3.4)	119.8	6.21 (d,3.3); 5.48 (d,3.3)	120.3	6.18 (d,1.8); 5.61 (d, 1.8)	121.8
14	1.40 (s)	16.2	5.26 (d, 1.3); 5.06 (d, 1.3)	109.7	1.08 (d, 7.6)	28.7
15	1.68 (d, 1.3)	17.4	4.89 (s); 4.81 (s)	112.7	1.18 (s)	22.7

a Recorded at 400 MHz, for ^1^H and 100 MHz, for ^13^C.

#### Compound 5

**FIGURE 2:**
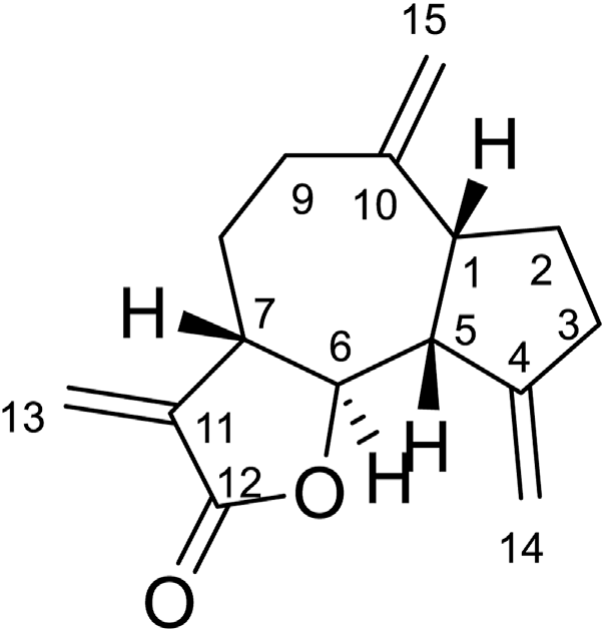
Chemical structural of dehydrocostus lactone.

Compound **5** was obtained as white amorphous powder. Its molecular formula was determined to be C_15_H_20_O_2_ based on the ESIMS data (m/z 233.1 [M + H]^+^), indicating six degrees of unsaturation. The ^1^H-NMR data (Table 6) showed three olefinic protons [*δ*_H_ 6.18 (1H, d, *J* = 1.9 Hz), 5.61 (1H, d, *J* = 1.6 Hz), 5.14 (1H, d, *J* = 4.1 Hz)]; one oxygen-bearing methine signal at *δ*_H_ 4.81 (1H, dt, 3.0, 6.5), three methine signals at *δ*_H_ 3.56 (1H, m), 2.43 (1H, m), and two methyl proton signal at *δ*_H_ 1.18 (3H, s), 1.08 (3H, d, *J* = 7.6 Hz),. The ^13^C-NMR spectrum (Table 6) contained fifteen carbon signals, which were assigned to one lactone carbonyl carbon (*δ*c 170.6), four olefinic carbons (*δ*c 149.2, 140.0, 121.8, 118.9), one oxy-methine carbon (*δ*c 76.6), two methine carbons (*δ*_C_ 39.6, 37.7), four methylenes (*δ*_C_ 42.8, 41.9, 32.8, 16.9), and two methyl carbons (*δ*_C_ 28.7, 22.7). These spectral data were elucidated to the published data of Alantolactone (Neves et al., 1999; Yuuya et al., 1999). Therefore, compound **5** was identified as dehydrocostus lactone and its molecular formula is shown in Figure 2. MS diagrams and ^1^H-NMR and ^13^C-NMR spectra were shown in Supplementary Figures S4–S6, respectively.

#### Compound 6

**FIGURE 3:**
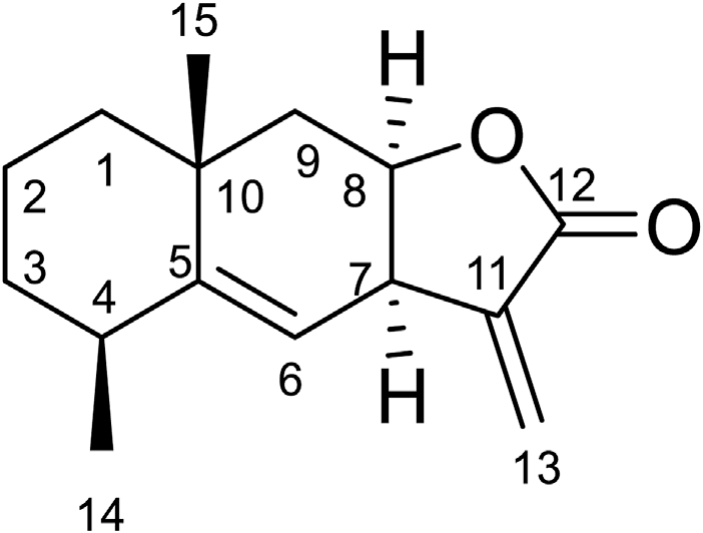
Chemical structural of alantolactone.

Compound **6** was obtained as white amorphous powder. Its molecular formula was determined to be C_15_H_20_O_2_ based on the ESIMS data (*m*/*z* 233.1 [M + H]^+^), indicating six degrees of unsaturation. The ^1^H-NMR data (Table 6) showed three olefinic protons [*δ*_H_ 6.18 (1H, d, *J* = 1.9 Hz), 5.61 (1H, d, *J* = 1.6 Hz), 5.14 (1H, d, *J* = 4.1 Hz)]; one oxygen-bearing methine signal at *δ*_H_ 4.81 (1H, dt, 3.0, 6.5), three methine signals at *δ*_H_ 3.56 (1H, m), 2.43 (1H, m), and two methyl proton signal at *δ*_H_ 1.18 (3H, s), 1.08 (3H, d, *J* = 7.6 Hz),. The ^13^C-NMR spectrum (Table 6) contained fifteen carbon signals, which were assigned to one lactone carbonyl carbon (*δ*_C_ 170.6), four olefinic carbons (*δ*_C_ 149.2, 140.0, 121.8, 118.9), one oxy-methine carbon (*δ*_C_ 76.6), two methine carbons (*δ*_C_ 39.6, 37.7), four methylenes (*δ*_C_ 42.8, 41.9, 32.8, 16.9), and two methyl carbons (*δ*_C_ 28.7, 22.7). These spectral data were elucidated to the published data of Alantolactone (Ming et al., 1989; Dereli et al., 2020). Therefore, compound **6** was identified as alantolactone and its molecular formula is shown in Figure 3. MS diagrams and ^1^H-NMR and ^13^C-NMR spectra were shown in Supplementary Figures S7–S9, respectively.

### Antifungal Activity of Active Compoundsactive Ingredients

Alantolactone, dehydrocostus lactone and costunolide isolated from extracts of Radix Aucklandiae had inhibitory effects on the spore germination of *F. graminearum*, *B. cinerea*, *C. gloeosporioides* and *F. oxysporum* (Table 7). The MIC values of alantolactone against *B. cinerea* and *F. graminearum* were 15.63 mg/L and 250 mg/L, and those against *C. gloeosporioides* and *F. oxysporum* were both more than 1,000 mg/L. The MIC values of dehydrocostus lactone against *F. graminearum*, *B. cinerea*, *C. gloeosporioides* and *F. oxysporum* were 3.91 mg/L, 62.25 mg/L, 125 mg/L, 250 mg/L, respectively. The MIC values of costunolide against *B. cinerea* and *F. graminearum* were 15.625 mg/L and 1,000 mg/L, and those against *C. gloeosporioides* and *F. oxysporum* were both more than 1,000 mg/L.

**TABLE 7:** MIC values of active compounds of Radix Aucklandiae against four plant-pathogenic fungi.

Treatments	MIC value (mg/L)			
	*F. graminearum*	*B. cinerea*	*C. gloeosporioides*	*F. oxysporum*
Alantolactone	250	15.63	>1,000	>1,000
Dehydrocostus lactone	62.25	3.91	250	125
Costunolide	1,000	15.625	>1,000	>1,000
Pyraclostrobin	40	2	1.25	2.5

## Discussion

Radix Aucklandiae is the dried root of *Aucklandia lappa* Dence. (Genus *Saussurea*, family Compositae). There are a few reports about the antimicrobial activity of the extract in medicine and agriculture. Research has reported that the extract has antimicrobial activity against *Helicobacter pylori* and *Streptococcus* in medicine (Yu et al., 2007; Han et al., 2011). The extract also has antimicrobial activity against *B. cinerea*, *A. alternata*, *Penicillium italicum* Wehmer, *Verticillium dahliae* and *F. oxysporum* (Hasi et al., 2009; Hu et al., 2009; Wang et al., 2012; Jin et al., 2019). In this study, extracts of Radix Aucklandiae had significant inhibitory effects on *B. cinerea*, *S. sclerotiorum* and *C. gloeosporioides*, and had obvious control effects on wheat powdery mildew, wheat head blight, strawberry grey mould and citrus anthracnose. Therefore, the extract has potential development and application value as a botanical fungicide.

The chemical composition of Radix Aucklandiae is diverse. At present, there have been many reports on the chemical composition of extracts of Radix Aucklandiae. For example, more than 200 compounds, such as sesquiterpene lactones, monoterpenes, phenylpropanoids, lignans, flavonoids and volatile oils, have been isolated from Radix Aucklandiae (Mao et al., 2017). In this study, three active compounds were isolated and purified from Radix Aucklandiae, and identified as alantolactone, dehydrocostus lactone and costunolide, which are all terpenoids.

At present, the biological activities of these three compounds have mainly been reported in the context of medicine and are less known in agriculture. In medicine, it was found that these compounds had antimicrobial, antitumor, anti-inflammatory, hepatoprotective and other pharmacological effects. These compounds can inhibit *Fusarium solani* (Mart.) Sacc., *Mycobacterium tuberculosis* and *Staphylococcus aureus* in the human body (Wahab et al., 1979; Cantrell et al., 1999; O’Shea et al., 2009). In agriculture, recent research has also shown that alantolactone has a significant inhibitory effect on *Phytophthora nicotianae* (Feng et al., 2018). Dehydrocostus lactone and costunolide have inhibitory effects on *Cunninghamella echinulata*, *Colletotrichum acutatum*, *B. cinerea* and *F. oxysporum* (Barrero et al., 2000; Wedge et al., 2000). In this study, alantolactone, dehydrocostus lactone and costunolide all had different degrees of inhibitory effects on *F. graminearum*, *B. cinerea*, *C. gloeosporioides* and *F. oxysporum*. Alantolactone have inhibitory effects on *F. graminearum* and *B. cinerea*, and costunolide have inhibitory effects on *B. cinerea*, while dehydrocostus lactone has inhibitory effects on four plant-pathogenic fungi. Among them, dehydrocostus lactone showed the best control effect on plant fungous diseases.

Dehydrocostus lactone is a guaiane-type sesquiterpene isolated from Radix Aucklandiae. Dehydrocostus lactone is mainly extracted by solvent extraction, microwave-assisted extraction, ultrasonic-assisted extraction, and separated and purificated by column chromatography (He et al., 2009; He et al., 2010). There are still difficulties in large-scale extraction. At present, the research on the structural modification and derivation of dehydrocostus lactone mainly focuses on Michael addition reaction at C-13 site and some oxidation reactions (Qian et al., 2012). The erivatives played an important role in tumor therapy. However, whether dehydrocostus lactone can be used as a lead compound in agricultural disease control remains to be verified. This study laid a foundation for the further development and utilization of extracts of Radix Aucklandiae as botanical fungicides.

## Data Availability

The raw data supporting the conclusions of this article will be made available by the authors, without undue reservation.
