# Supplementary material for: Antimicrobial Effects and Active Compounds of the Root of Aucklandia Lappa Decne (Radix Aucklandiae)
Source: Front Chem. 2022 Apr 6;10:872480. doi: 10.3389/fchem.2022.872480 (PMC9019365; doi:10.3389/fchem.2022.872480)
Supplement: Supplementary file 1 [file DataSheet1.docx]

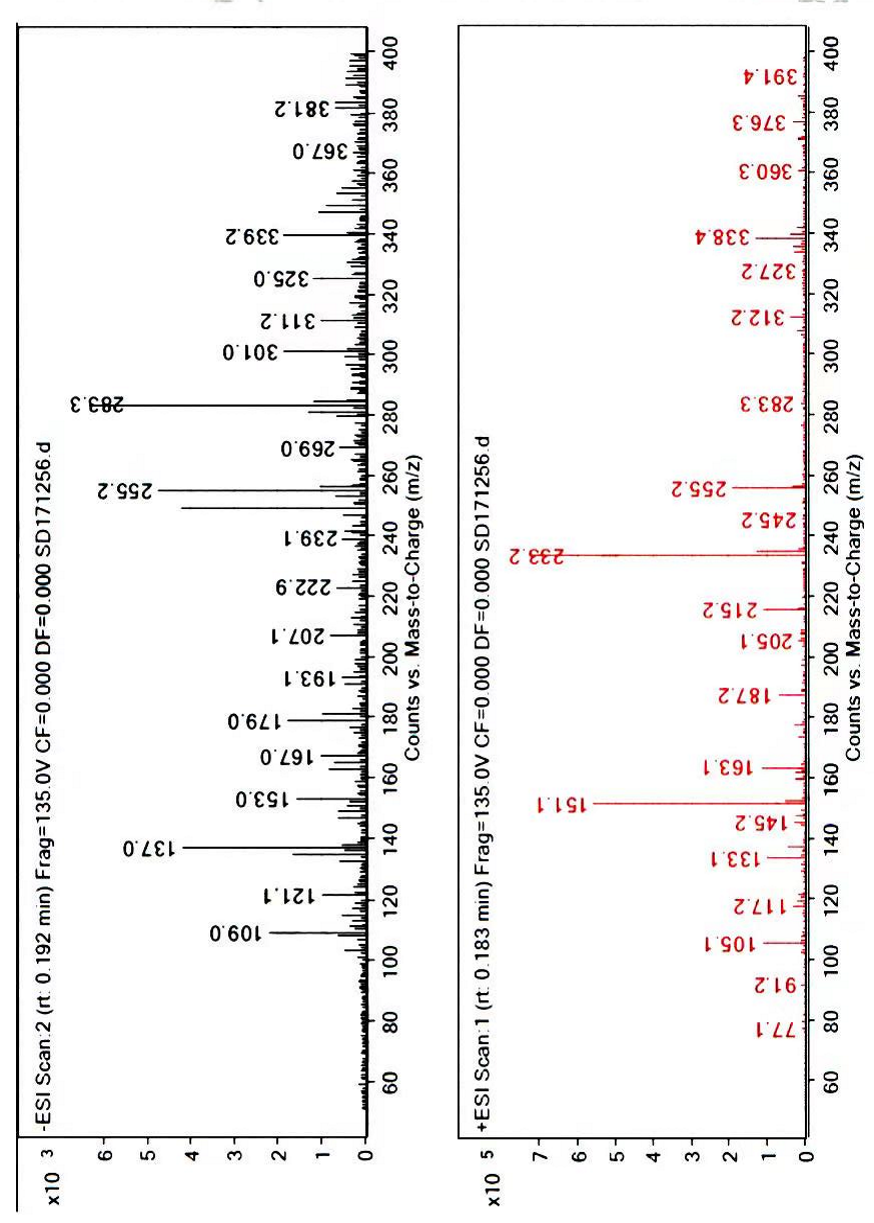


Figure S1 MS diagram of the Compound **2**


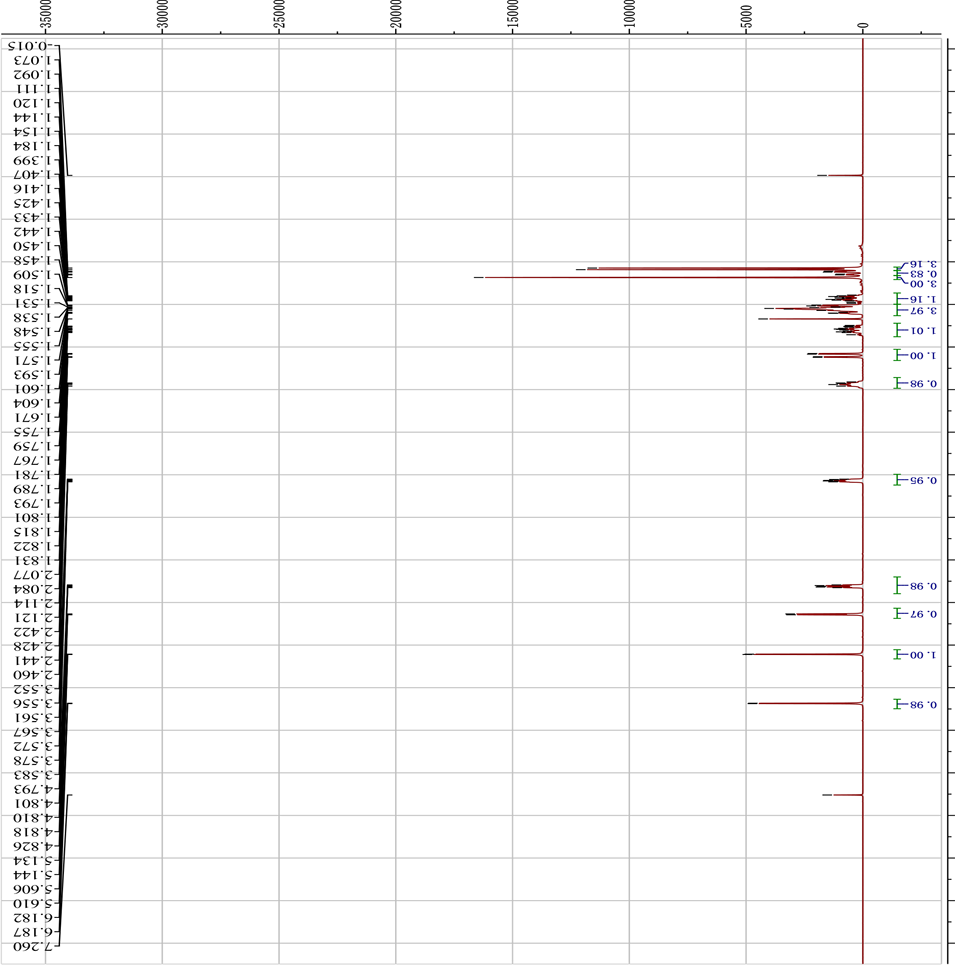


Figure S2 ^1^H-NMR spectra of Compound **2**


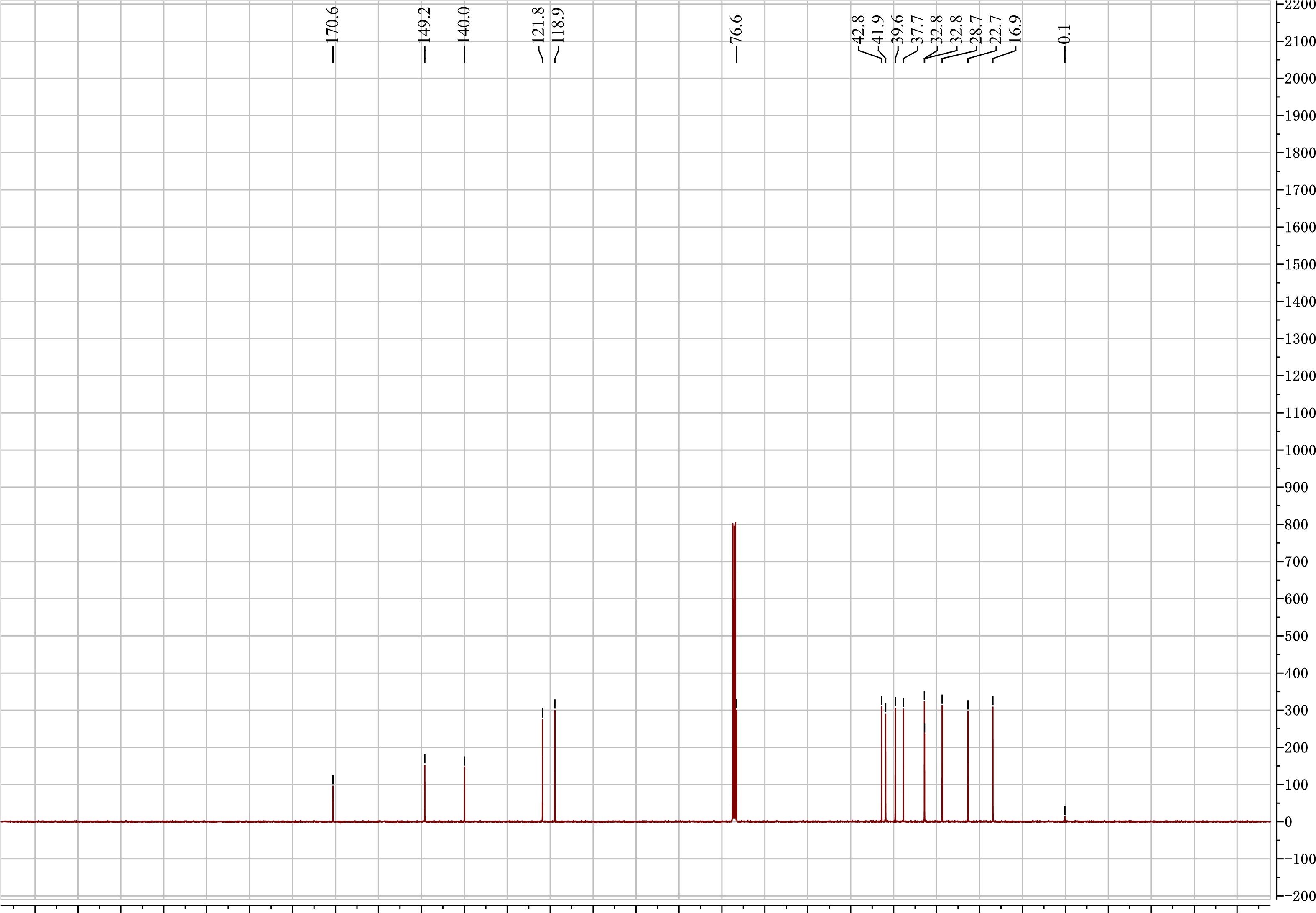


Figure S3 ^13^C-NMR spectra of Compound **2**


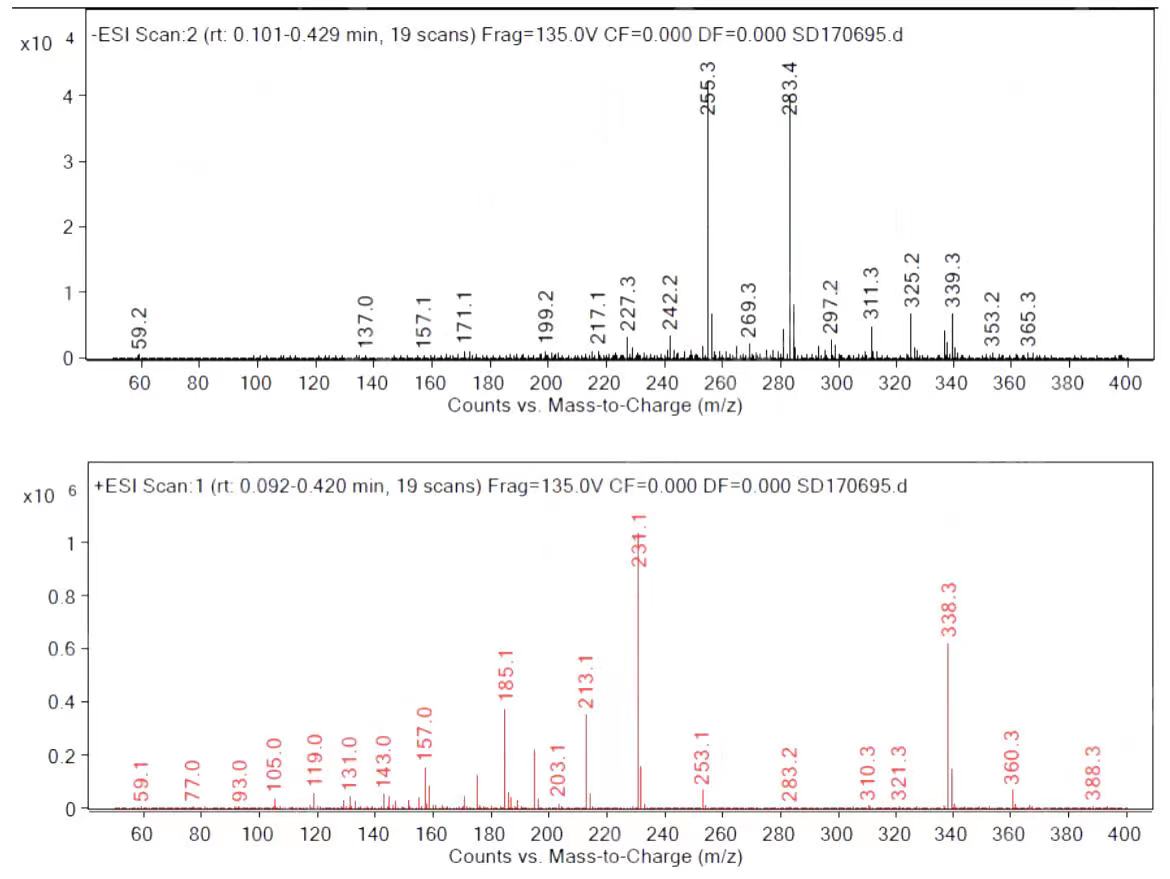


Figure S4 MS diagram of the Compound **5**


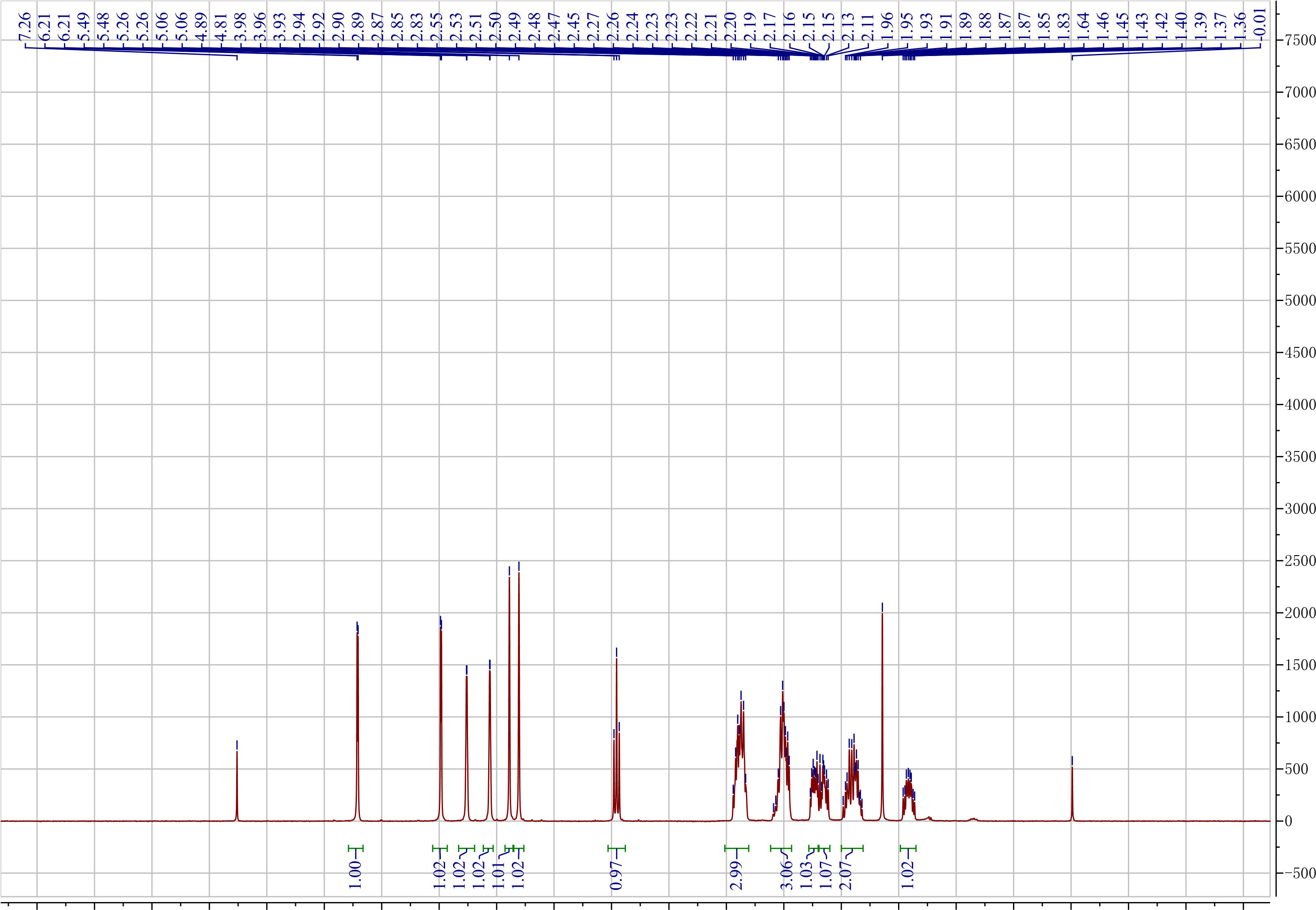


Figure S5 ^1^H-NMR spectra of Compound **5**


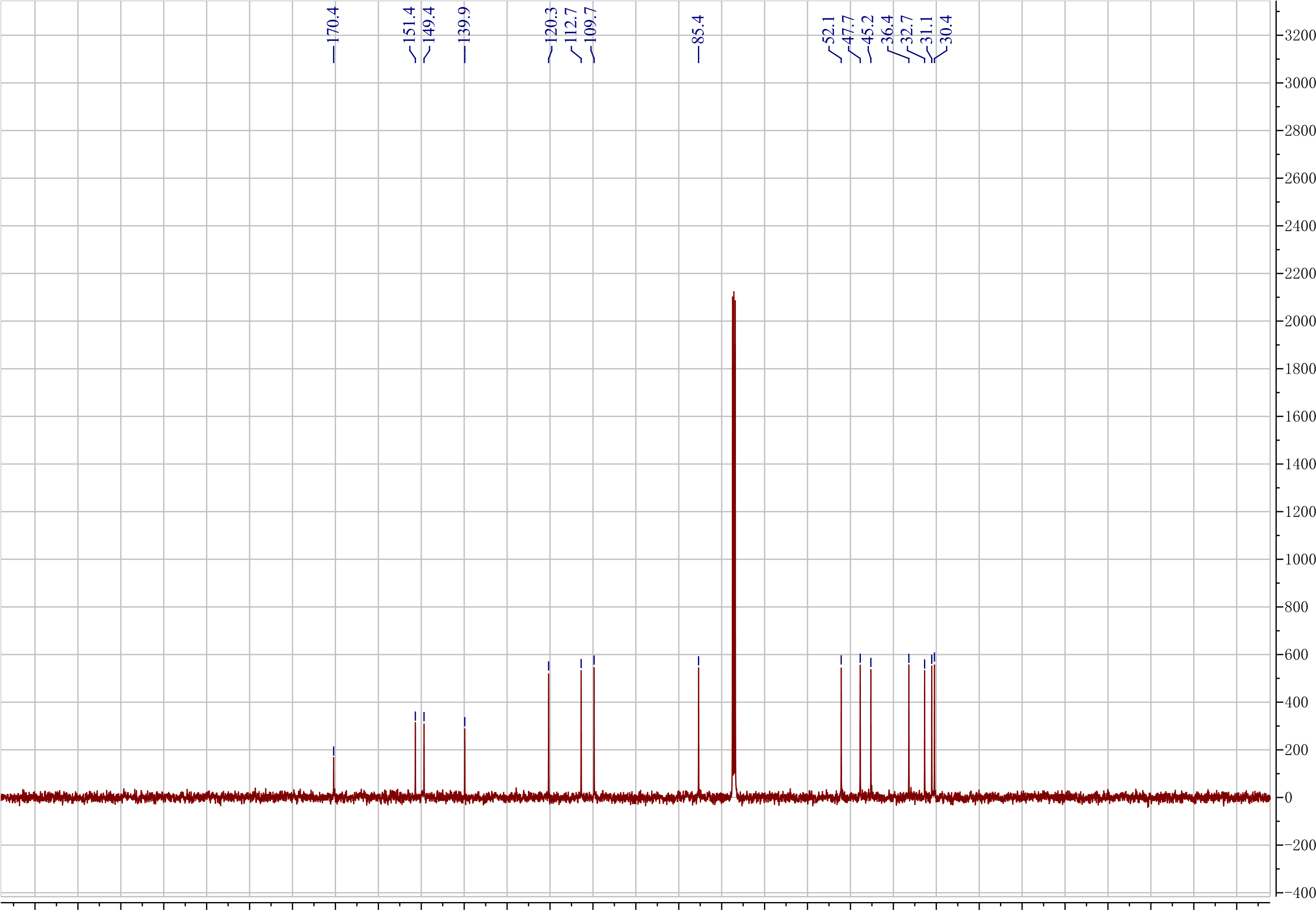


Figure S6 ^13^C-NMR spectra of Compound **5**


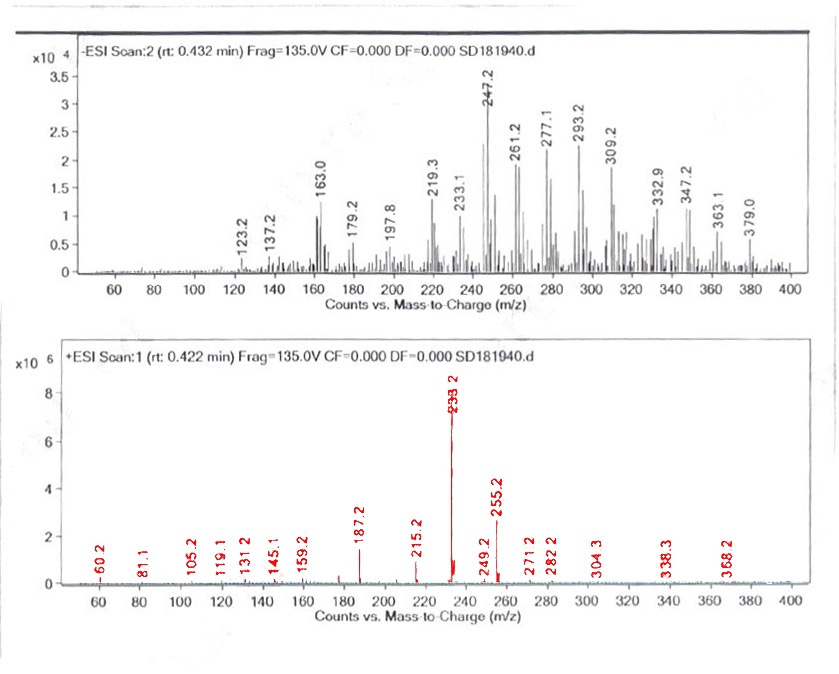


Figure S7 MS diagram of the Compound **6**


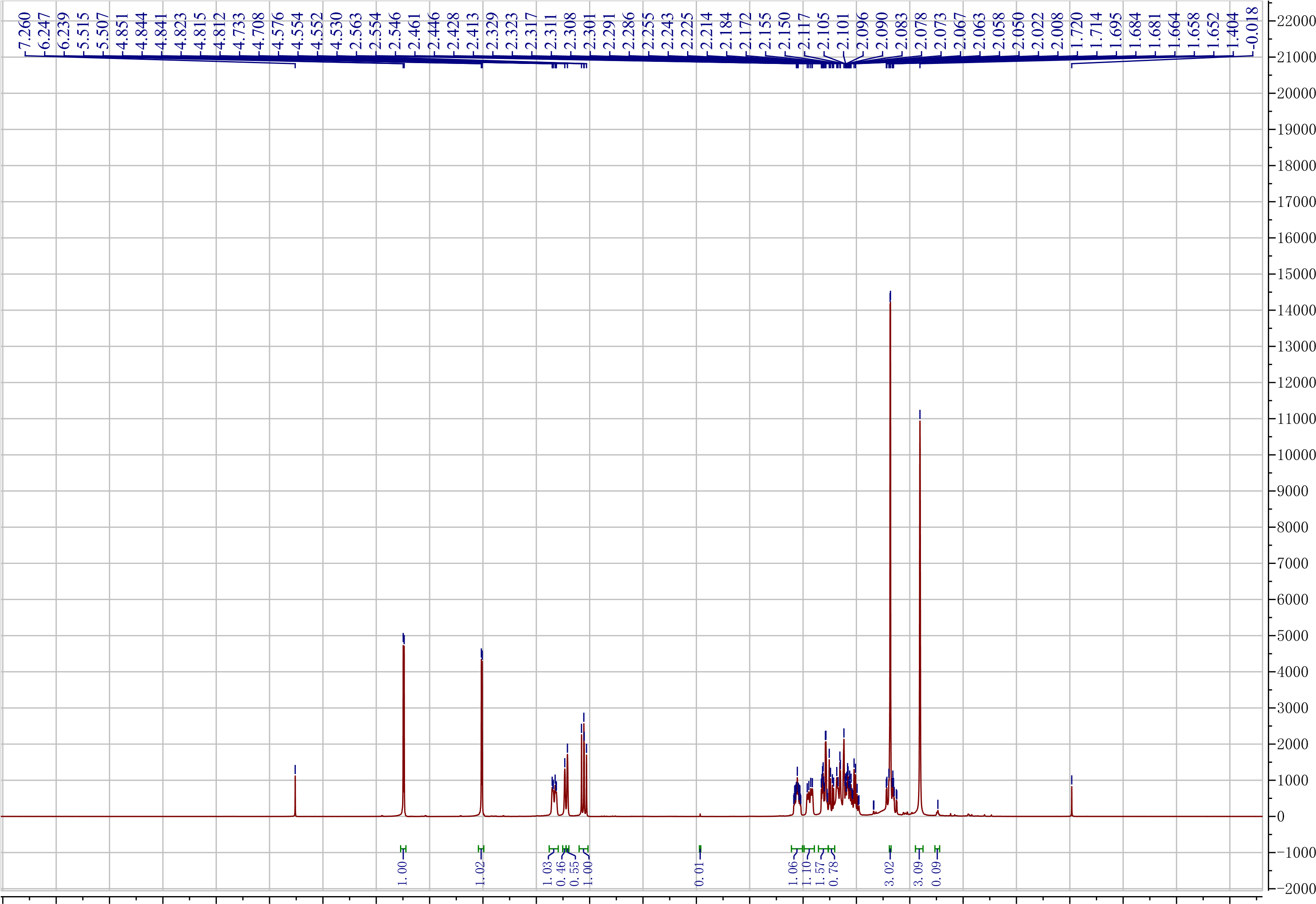


Figure S8 ^1^H-NMR spectra of Compound **6**


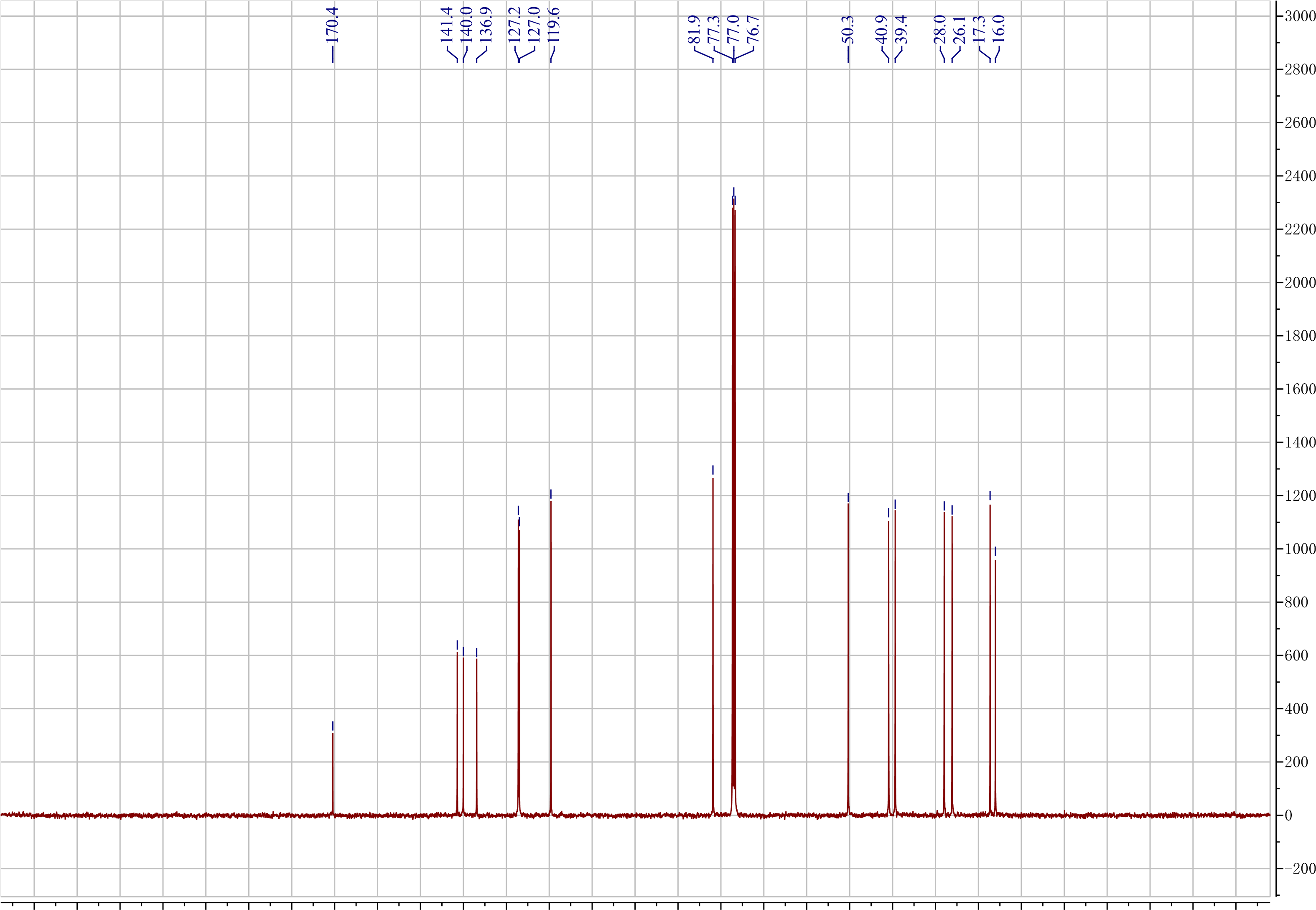


Figure S9 ^13^C-NMR spectra of Compound **6**
